# Motivational stimuli to donate sperm among non-donor students

**DOI:** 10.1186/s12610-023-00201-2

**Published:** 2023-10-17

**Authors:** Maya Ronen, Alon Kedem, Sarit Avraham, Michal Youngster, Gil Yerushalmi, Ariel Hourvitz, Itai Gat

**Affiliations:** 1 Faculty of Medicine, Tel Aviv University, Tel Aviv, Israel; 2 Shamir Medical Center, Zrifin, Israel; 3 IVF Department, Shamir Medical Center, Zrifin, Israel; 4 Sperm Bank & Andrology Unit, Shamir Medical Center, Zrifin, Israel

**Keywords:** Sperm donation, Sperm bank, Identity disclosure, Anonymity, Don de sperme, Banque de sperme, Divulgation d'identité, Anonymat

## Abstract

**Background:**

Sperm banks face a continuously evolving gap between the increasing demand for sperm donation (SD) vs. limited available reserve. To improve donors’ recruitment and increase supply, motivations towards SD should be investigated specifically among young men who have the potential to become donors. Our aim was to evaluate factors which increase and decrease predisposition to donate sperm among non-donor students, who represent a “potential pool” for possible donors’ recruitment.

**Results:**

Ninety-three men fulfilled the questionnaire with mean age of 28.2 ± 4.5 years. The most powerful incentive to donate sperm was financial reward followed by a willingness to help others to build a family (3.8 and 3.4, respectively). The most dominant consideration to decline donation was the fear of anonymity loss and future regret (4 and 3.8). While participants’ willingness for anonymous SD was fair (2.8), the open-identity donation was rated significantly lower (1.75, *p* < 0.01). Familiarity with recipients and offspring had lower scores (1.9–2.2) as well.

**Conclusions:**

Young single men represent a suitable cohort for anonymous donation. Financial reward and willingness to help others are important positive incentives while anonymity preservation is crucial to maintain their willingness towards SD. Regulatory shifting towards open-identity SD necessitates the establishment of an alternative “potential pool” population as a reliable source to recruit donors.

**Supplementary Information:**

The online version contains supplementary material available at 10.1186/s12610-023-00201-2.

## Résumé

**Contexte:**

Les banques de sperme font face à un écart en constante évolution entre la demande croissante de dons de sperme et les réserves disponibles limitées. Pour améliorer le recrutement des donneurs et augmenter l'offre, les motivations à l'égard du don de sperme devraient être étudiées spécifiquement chez les jeunes hommes qui ont le potentiel de devenir donneurs. Notre objectif était d'évaluer les facteurs qui augmentent et diminuent la prédisposition au don de sperme chez les étudiants non-donneurs, qui représentent un « bassin potentiel » pour le recrutement possible de donneurs.

**Résultats:**

Ninety-three hommes ont rempli le questionnaire avec un âge moyen de 28,2±4,5 ans. L'incitation la plus puissante à donner du sperme était la récompense financière, suivie de la volonté d'aider les autres à fonder une famille (3,8 et 3,4, respectivement). La considération la plus dominante pour refuser le don était la peur de perdre l'anonymat et les regrets futurs (4 et 3.8). Alors que la volonté des participants de faire un don anonyme de sperme était passable (2,8), le don d'identité ouverte a été jugé significativement plus faible (1,75, p<0,01). La familiarité avec les receveurs et la progéniture avait également des scores plus faibles (1,9-2,2).

**Conclusions:**

Les jeunes hommes célibataires représentent une cohorte appropriée pour le don anonyme. La récompense financière et la volonté d'aider les autres sont des incitations positives importantes, tandis que la préservation de l'anonymat est cruciale pour maintenir leur volonté de donner du sperme. L'évolution réglementaire vers le don de sperme à identité ouverte nécessite l'établissement d'une population alternative de « bassin potentiel » comme source fiable pour recruter des donneurs.

## Background

Since the introduction of sperm donor insemination in 1884 and over the following decades, sperm donation (SD) was performed in secrecy for heterosexual couples who suffered from male subfertility [1]. Over the last decades, improvements in fertility treatments for male infertility combined with evolving novel family structures led to fundamental changes in SD. Current patients’ population includes mainly single and lesbian women, with rising new demands and challenges. Families formed through donated gametes or embryos represent a unique population with special considerations, including health, social, and emotional outcomes [2]. Most offspring of SD are aware of their paternal biologic origin, raising questions regarding psychological aspects such as identity development [3, 4]. Legislation of SD varies between different countries and societies. Some topics are still far from being consensual such as SD to same-sex couples [5], birth limitation per donor and the innate conflict between offspring’s rights [6], interests and need for identifying information versus preserving donor’s anonymity [7].

Sperm banks (SB) face increased demand for SD by the growing population of single women and same-sex couples combined with expanding restrictions over donor recruitment and activity [8]. To maintain adequate supply in these challenging circumstances, perceptions and attitudes towards SD should be characterized. Sperm donors, who are the core factor throughout SD, are obviously affected by the developing attitudes and changing legislation. For example, donation permission from single only vs. possibly married men and shifting from anonymity to open-identity donation result in different donors’ characteristics [9–11]. Previous research has almost exclusively focused on donors’ and their ex-post rationalization of their decision-making process [12]. While the importance of focusing on sperm donors is obvious and fundamental, these findings do not reflect general sociological perceptions and motivations toward SD. While such studies inform an understanding of both positive and negative correlates of donation behavior and its possible drivers, they provide little insight into the preferences, experiences, understanding, and decision processes of those yet to donate [12]. Unfortunately, studies of non-donor men are limited. In Their review, Van den Broeck et al. reported that only a single study out of 29 focused on non-donors [13]. However, that study [14] focused on medical students only; therefore, the ability to generalize its findings to other populations is limited. Donors and intended donors represent populations far from general or non-donor men. Understanding men’s perceptions about sperm donation might help achieve two goals: first, to determine whether measures are needed to increase the acceptance of sperm donation, and second, to find out what adaptations are required to create more efficient recruitment campaigns [15].

A prominent controversial aspect of SD lies in the conflict between donors’ interest in preserving their anonymity vs. identity disclosure. Over the past decade, evolving literature describes the growing interest of offspring (especially those in single-parent family opposed to heterosexual couples) to know their donor’s identity [16–18]. However, shifting from anonymous to identity disclosure may result in a temporarily severe shortage of donors since most anonymous donors will cease donation [19, 20]. Legislation shift either through traditional sperm bank track or online may result in novel donor characteristics [8, 21]. The evolving ‘introduction websites’ and social media forums outside of clinical (formal) settings supply alternative pathways for sperm donation. Harper et al. categorized women use to three settings: those who want to have a child with no further involvement of the donor; those who wish to know the identity of the donor from the start; and those who intend to electively co-parent, that is, to bring up the child together with the donor/father. On the other hand, these “informal donors” are more likely to be in some form of committed relationship and more likely to identify as a sexuality other than heterosexual [22, 23].

In summary, long term sociological trends combined with evolving perceptions and demands from SB necessitate evaluating not only actual donors and candidates but also non-donors men from a relevant demographic background (ex., students, young single) – a potential pool for donors’ enrolment. In addition to positive and negative stimuli toward sperm donation, a specific focus should be implemented on identity disclosure vs. anonymity. The current study aimed to investigate these motivational stimuli toward sperm donation among non-donors Israeli students, who represent the potential population for sperm donation recruitment.

## Methods

### Population

The current research is a part of a larger study conducted at Tel Aviv University, Israel, focused on general population perceptions towards sperm donation measured by an anonymous digital questionnaire between January-February 2021 (Gat: Students' perceptions regarding sperm donation: dilemmas reflections with dominant demographic effect – manuscript submitted for publication, 2023). The study included only non-donor male students who replied to a specific questionnaire’s section focused on motivational aspects related to SD. The study’s methodology has been described previously (Gat: Students' perceptions regarding sperm donation: dilemmas reflections with dominant demographic effect – manuscript submitted for publication, 2023). Briefly, applications to participate in the study were published in closed groups of students at Tel Aviv University on social media platforms, including Facebook and WhatsApp. The application included a short statement regarding the sake of the study and a special digital link for those who responded to the questionnaire. Students who clicked on the link were referred immediately to the questionnaire, which was digitally implemented using QuestionPro system – an accepted open-access software designed for such applications. Then, before initiating the study, a further announcement was presented declaring that by clicking over the next bottom to enter the questionnaire, the participant declares consent to participate in the study anonymously. Only participants who answered more than 80% of the questions were included. Participants who donated sperm previously were excluded.

### Study questionnaire

The questionnaire included 30 questions divided into three main sections: (1) demographic data including 9 close-ended questions (age, marital status, etc.) and prior acquaintance with SD and people involved (donors and recipients); (2) 6 multiple choice questions aimed to evaluate prior knowledge regarding sperm donation; (3) research’s main section composed of 5 positive and 5 negative stimuli towards willingness to donate sperm followed by 5 questions focused on the impact of identity disclosure versus anonymity. Participants were asked to rate each question 1–5 on a Likert scale.

In their review focused on SD perceptions, Van den Broeck et al. observed that 23/25 questionnaires were constructed specifically for that topic without psychometrical validation [13]. Since scientific literature regarding sperm donation already includes diverse questionnaires and statistical validation for a completely new questionnaire is practically impossible, we decided mainly to rely on previously used questions [24–27] with specific additions and adaptations for the current population.

Prior to initiating the study, the questionnaire was sent to 20 non-anonymous students (ages 23–40) to test the digital platform’s comfortability, drop-out rate, and questions clarity. That pre-test resulted in rephrasing a few questions and minor adjustments to limit response duration to 6 min to reduce the drop-out rate, resulting in the final questionnaire version (Supplement 1).

### Statistical analysis

Demographic data collected by the first questionnaire’s section was initially descriptive only. The second section of informative multiple-choice questions was evaluated per corrected answers (how many answered correctly single vs. two, three questions, etc.), followed by a total grade per participant on a 0-100 points scale with every question getting 16.67 points.

The study primary outcome was the main third questionnaire section. We persisted in using similar phrasing within each component - positive and negative stimuli questions; anonymous vs. open-identity; and familiarity with recipients and offspring. Participants ranked each stimulus on 1–5 Likert scale, summarized as mean for each stimulus. Consequently, Wilcoxon test was used to compare means between five positive and five negative stimulations. *P* value < 0.05 was regarded as statistically significant.

## Results

### Population demographic characteristics

**Fig. 1:**
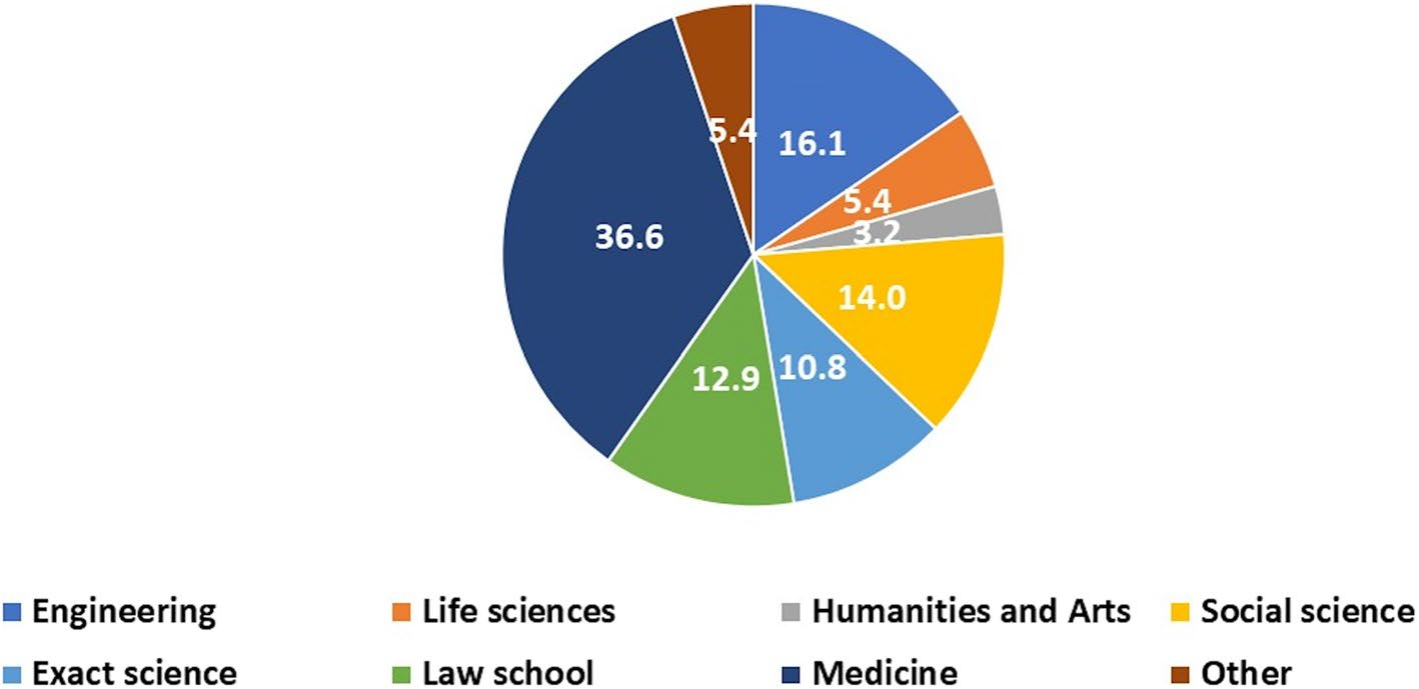
Students’ faculties (q. 6). Study population included participants from diverse faculties. Most common was medical school followed by engineering and social sciences

The current study included 93 male students. The mean age was 28.2 ± 4.5 years. 68 (73.1%) participants were single compared to 25 (26.9%) married and 1 (1.1%) divorced. 75 (80.6%) participants had no children opposed to 18 (19.4%) fathers. 78 (83.9%) defined themselves as secular while 10 (10.8%) as religious and 6 (6.3%) as traditional. From academic perspective, 50 students (53.7%) had been allocated to Bachelor’s degree. Students’ faculties are described in Fig. 1. Focusing on previous acquaintance with sperm donation, 18 (19.4%) had personal familiarity with women who used sperm donation, and 42 (45.2%) heard about sperm donation from public media only. 14 students (5.5%) considered donating but avoided it eventually.

**Fig. 2:**
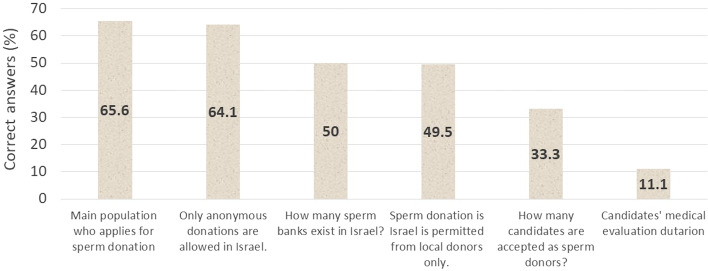
Participants’ previous knowledge (q. 10-15). Six questions were introduced to assess previous knowledge regarding SD. While most participants answered correctly that single women are main population who apply for SD and optional non-anonymous donation in Israel, only 11.1% knew that medical assessment lasts more than 5 months

Participants’ previous knowledge examination was performed by six informative questions (numbered 10–15, Supplement 1). Upon four questions, half participants or more answered correctly, especially regarding the main recipients’ population and anonymous donations (65.6% and 64.1% corrected answers, respectively). Only 11.1% answered accurately regarding the duration of the candidates’ evaluation (Fig. 2).

### Sperm donation’s motivational stimuli

**Fig. 3:**
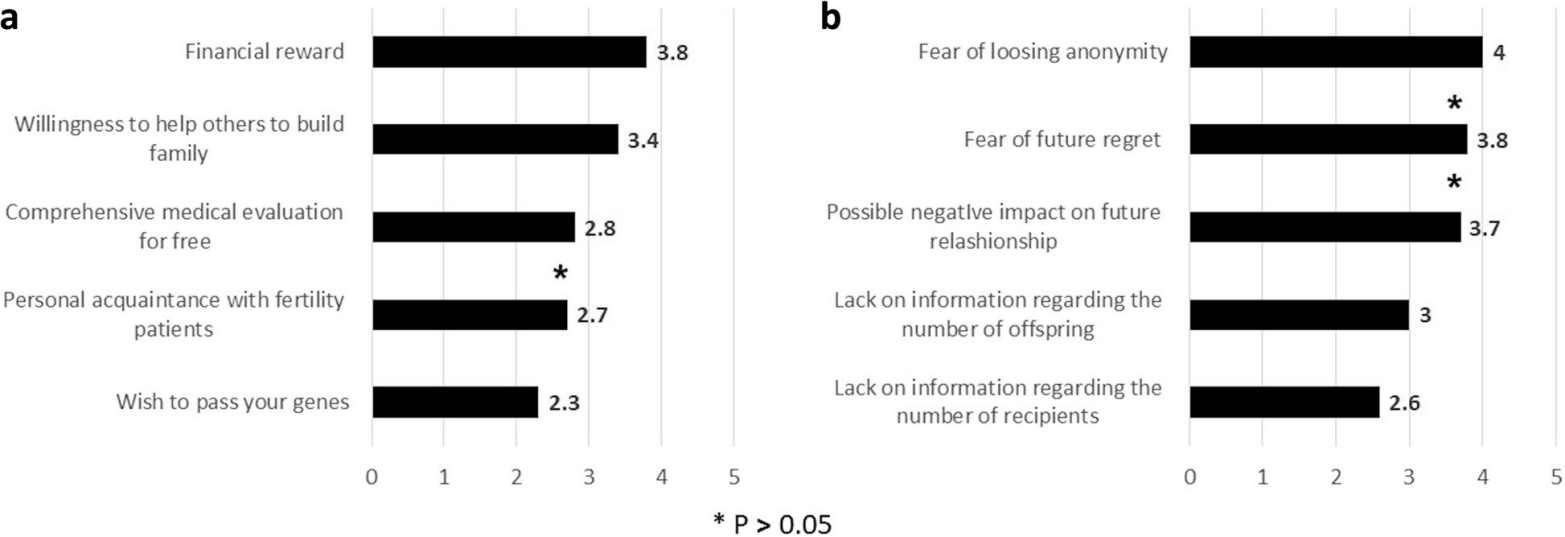
Positive and negative stimuli towards sperm donation. **a** Positive stimuli comparison (q. 18-22). Participants ranking using 1-5 Likert scale was summarized as mean for each stimulation. Positive stimulus comparison by Wilcoxon test resulted with significant grading differences between all stimuli (*p*<0.05) except similar ranking to “comprehensive medical evaluation for free” and “personal acquaintance with fertility patients”. Financial reward had the highest rank followed by willingness to help others. Passing genes to future generation was ranked as lowest. **b** Negative stimuli comparison (q. 23-27). Participants ranking using 1-5 Likert scale was summarized as mean for each stimulation. Negative stimulus comparison by Wilcoxon test resulted with significant grading differences between most stimuli (*p*<0.05) Among aversive stimuli, fear of losing anonymity and future regret (4 and 3.8 on 1-5 Liker scale, respectively) were significantly highest ranked compared to all other statements. Possible negative impact on future relationship had slightly lower rank (3.7)

The primary endpoint of the present study was the third and central section of the questionnaire. Each statement \ stimuli was ranked using 1–5 Likert scale and the mean was used for comparison. Among **positive** motivational stimulation, financial reward tallied the highest mean calculated on 1–5 Likert scale (3.8), followed by altruism and free medical evaluation (3.4 and 2.8, respectively), while the lowest score was given to the wish to pass genes onto the next generation (2.3). On the other hand, the most **aversive** factor was the fear of anonymity loss and future regret (4 and 3.8, respectively), followed by the possible negative impact on the future relationship and family (3.7)., Almost all scores were significantly different throughout both positive and negative stimulations’ means comparisons (Fig. 3).

**Fig. 4:**
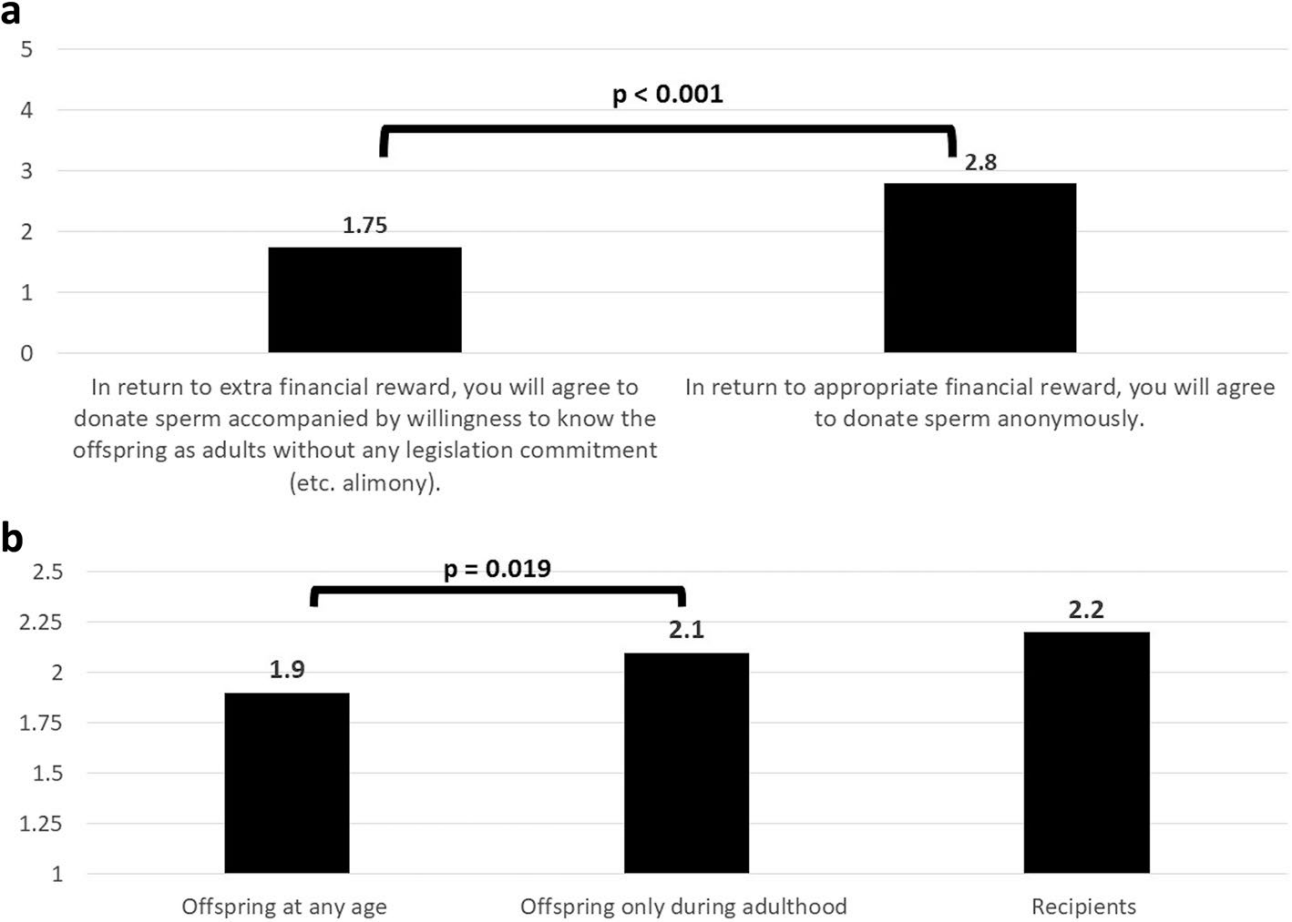
Anonymity versus identity disclosure. **a** Willingness for identity disclosure (q. 16-17). Non donor students were asked specifically regarding their willingness to donate sperm either anonymously for “financial reward” or alternatively as identity disclosure donors without any legislation commitment for “extra financial reward”. First choice was ranked significantly higher (mean 2.8 vs. 1.75 on 1-5 Likert scale, *p*<0.001). **b** Willingness for recipients and offspring familiarity for extra payment (q. 28-30). Focusing on possible familiarity with offspring on recipients “for extra payment”, participants declared significantly low willingness towards young age offspring

Two attributes examined the aspect of identity disclosure. First, we asked participants to what extent they would consider anonymous sperm donation and open identity donation in return for an extra payment. While anonymous donation was rated as an acceptable option (mean 2.8), the non-anonymous track scored significantly lower (mean 1.75, *p* < 0.001). Second, participants were required to rate their attitudes towards familiarity with recipients and offspring. Acquaintance offspring at any age was rated significantly lower than during adulthood (mean 1.9 vs. 2.1, respectively, *p* = 0.019). While acquaintance with mothers was higher than with adult offspring, that difference was not statistically significant (Fig. 4).

Demographic characteristics, including an academic degree or faculty, have not been associated with significant findings regarding motivational stimuli throughout the questionnaires.

## Discussion

Over the past decades, sociological changes and medical developments forced fundamental adaptations within SBs’ activities. What was once taboo has become a topic for public discussion. Contrary to the past, modern offspring from SD are aware of their mode of conception, resulting in thoughtful ethical dilemmas affecting sperm donors’ recruitment and SD implementation. The current research, which evaluated non-donors’ motivations, highlights the importance of financial reward and altruism as strong motivations for SD, like previous report among non-donor students [15]. Interestingly, although the desire to help others build a family was highly rated, participants expressed very low scores regarding their willingness to familiarize with recipients and offspring. These findings are compatible with various previous reports which emphasized the combination of altruism and anonymity [13, 28]. We assume that participants age and marital status (single) may have strong impact on these preferences. Further studies using psychological methodologies (ex. interviews and questionnaires) may supply deeper understating for these motivations.

Similar characteristics have been reported among donors from other countries and societies. Previous review identified four different types of motivation for SD - altruism, financial compensation, procreation or genetic fatherhood, and finally questions about the donor’s own fertility [13]. Although all factors seem relevant among non-donors in the present study, financial reward and altruism are mostly dominant. Mahieu et al. [20] have reported that only 20.1% of Belgian donors would continue to donate in case of identifiable donation. Another important finding was that those less interested in recipients and offspring would discontinue donating in case of anonymity cessation. Similarly, proportion of anonymous Danish donors who would stop their donations if anonymity was abolished has been reported as 51- 67% between those who donated sperm between 1992 and 2012, respectively [19]. Although current study included non-donor young men, our findings are very similar to those observed among actual donors: highest priority of anonymity and low interest regarding offspring familiarity.

Sweden was the first country to enable only identity-disclosure sperm donation in 1985. Since then, there has been a steady increase in the number of countries and jurisdictions that are revoking the use of anonymous sperm donors [29]. While some reports focused on donors shortage, leading to “reproductive traveling” to other countries, which enable anonymous SD [8], others have reported similar donor supply [30] but different characteristics [21]. These differences may vary from diverse cultural and sociological differences between societies. For example, more than half Danish donors declared they would stop donation once anonymity would be prohibited [19], same is true only among 29% American donors while the majority would continue donating for extra financial reward of 60$ per donation [31]. We assume these differences arise from different balance between donors’ consideration based on financial reward vs. altruism. Future comparisons between societies may shed a light on that aspect. Israeli regulations differentiate between local anonymous donors and imported donors from Europe and USA who may be anonymous or open identity. The present results emphasize anonymity’s crucial role in preserving students’ readiness to consider SD. Not only that, extra financial reward was not sufficient to maintain similar willingness for SD with identity disclosure, and acquaintance with women and offspring (especially young) got low scores (1.9–2.2), demonstrating its aversive impact on present study population. Consequently, even those who believe that donors’ identity disclosure is inevitable should establish a reliable alternative source for donors’ recruitment. Optional assumption that single men (either hetero or homosexual) may be candidates for recruitment assuming that open identity donation may be appropriate compensation rather than conventional family deserves further investigation. Since donors’ identity disclosure remains important for 23–58% among offspring [16], we suggest maintaining two parallel of both directives for SD – anonymous and disclosed identity.

The fundamental role of anonymity to maintain donors’ supply raise further conflict related to genetic testing expansion. By 2016, over 3 million people have already used direct-to-consumer genetic testing to find information about their ancestry, and many are participating in international genetic genealogy databases that will match them with relatives. These commercially available genetic kits enable individuals to seek their relatives without mediators, raising serious concerns regarding the ability to maintain donors’ crucial anonymity. Therefore, donors should be informed that their anonymity is not guaranteed, as they may be traced if their DNA, or that of a relative, is added to a database [32, 33].

Most of the existing literature regarding donors’ incentives focused on actual donors and candidates who applied for SB to become donors. Such studies are biased toward the existing donors’ population, which is inclined toward single young men [13]. However, acceptance as a sperm donors and maintaining stable donors’ supply relies on “potential pool” population– an identifiable fraction of the general population with certain demographic characteristics suitable for local legislation SD requirements. Therefore, the present study focused on non-donors’ students’ population – a potential source donor recruitment. It should be noted that 83.9% of participants classified themselves as secular, which are more liberal towards SD compared to conservative and religious beliefs [34, 35]. Recently, Whyte et al. published Australian study focused on general population approached via social media using anonymous online survey. Gender comparison resulted by significant differences related to conditional willingness, barriers, unconsidered and conscientious objector [12]. That pioneering research emphasizes the importance of non-donor studies to get better understanding and characterization, which may lead to improved recruitment strategies to fulfill gamete donor shortage. However, current study focused specifically on men demonstrating clear picture of positive and aversive stimuli towards SD. Participants’ willingness to consider SD under current regulations (anonymous donation) of 2.8 was quite open-minded, confirming our preliminary hypothesis regarding that population. Most donors arise from certain marital and age groups (young single men) motivated mainly by the financial reward and altruism that apply to an anonymous donation [10, 21]. The shift towards older, married, and more altruistic motivational populations in countries with open-identity donation such as Sweden compared to higher rate of young single anonymous donors such as Denmark suggests a different recruitment pool [8, 19].

### Limitations of the study

The main contribution of the current study was the inclusion of non-donor men. To get relevant assessment for the sake of the study, we focused on students’ population who share similar demographic characteristics as sperm donors (young single men) rather than applying for general population research. However, that specific inclusion perspective is also prominent limitation of the study since students’ population has specific characteristics (ex. low income, special interest among recipients towards highly educated donors [36]. which prevent generalization of our findings to general population. Older married men stimuli towards SD differ significantly including key points such as perceptions towards anonymity [21]. Furthermore, perceptions of married donors’ spouses may be very interesting topic for investigation in future studies. Additional limitation relies on gender differences [12]; Current data should not be generalized to women and potential egg donors, who require specific different research.

## Conclusions

The strongest motivational stimuli towards SD among non-donor student population are financial reward and altruism. Several perspectives demonstrated the crucial importance of donors’ anonymity preservation: (1) Fear of losing anonymity was the leading cause to refrain from donation; (2) suggestion towards identity disclosure donation in return for extra payment was rated significantly lower than anonymous donation and (3) lowest score demonstrated towards communication with offspring, especially before adulthood. As demand for SD continuously grows and involves medical aspects that limit donors’ supply, the preservation of anonymous donations is crucial. Open-identity donations have important advantages from an offspring perspective. Therefore, efforts should be made to characterize and establish a suitable “potential pool” for such potential donors.

### Supplementary Information

**Additional file 1: Supplement 1.** Research questionnaire.

## Data Availability

The study’s data is available upon request.

## Abbreviations

SD: Sperm donation
SB: Sperm banks
