# Supplementary material for: Motivational stimuli to donate sperm among non-donor students
Source: Basic Clin Androl. 2023 Oct 17;33:29. doi: 10.1186/s12610-023-00201-2 (PMC10580632; doi:10.1186/s12610-023-00201-2)
Supplement: Supplementary file 1 — Additional file 1: Supplement 1. Research questionnaire. [file 12610_2023_201_MOESM1_ESM.docx]

**Supplement 1: Research questionnaire**

**Demographic data**

1. Gender.
2. Age.
3. Marital status.
4. Number of children.
5. Education.
6. University faculty.
7. Religiosity.
8. Socioeconomic status.
9. What is your personal acquaintance with sperm donation?

**Previous knowledge regarding sperm donation (corrected answers are in bold):**

1. How many sperm banks exist in Israel? (1-5 \ 6-10 \ 11-15 \ **>15**)
2. How many male applicants are eventually accepted to become sperm donors? (**<20%** \ 20-40% \ 40-60% \ > 60%)
3. How long does it take (in months) to complete the medical evaluation of a sperm donation candidate, starting from his initial application until final approval as a sperm donor? (>1 \ 1-3 \ 3-5 \ **5 <**)
4. Only anonymous donations are allowed in Israel. (right \ **wrong**)
5. Sperm donation is Israel is permitted from local donors only. (right \ **wrong**)
6. Which is the main population who applies for sperm donation? (**Single women** \ same sex couples \ heterosexual couples)

**Motivational Stimuli Towards Sperm Donation**

**Please rate the following statements as follows: 1 - Strongly disagree; 5 - Strongly agree**

1. In return to appropriate financial reward, you will agree to donate sperm anonymously.
2. In return to extra financial reward, you will agree to donate sperm accompanied by willingness to know the offspring as adults without any legislation commitment (etc. alimony).
3. Willingness to help other to build their family is an important stimulation to donate sperm.
4. Wish to pass your genes to next generation is an important stimulation to donate sperm.
5. Personal acquaintance with fertility patients is an important stimulation to donate sperm.
6. Comprehensive medical evaluation (such as fertility capability, genetic tests, sex transmitted diseases etc.) for free is an important stimulation to donate sperm.
7. Financial reward is an important stimulation to donate sperm
8. Fear of anonymity loss (ex. in social media) reduces the willingness to donate sperm.
9. Fear of possible harm to future relationships and family reduces the willingness to donate sperm.
10. Fear of future regret reduces the willingness to donate sperm.
11. Lack of information regarding number of recipients reduces the willingness to donate sperm.
12. Lack of information regarding number of offspring reduces the willingness to donate sperm.
13. Optional acquaintance with recipients in return to extra financial reward may improve the willingness to donate sperm.
14. Optional acquaintance with offspring at any age in return to extra financial reward may improve the willingness to donate sperm.
15. Optional acquaintance with offspring only during adulthood in return to extra financial reward may improve the willingness to donate sperm.
